# National variation in the treatment of lung cancer in a universal healthcare context

**DOI:** 10.1038/s41416-025-03071-9

**Published:** 2025-06-06

**Authors:** Jason Gurney, Anna Davies, James Stanley, Alex Dunn, Laird Cameron, Shaun Costello, Paul Dawkins, Jonathan Koea, Jesse Whitehead, Christopher GCA Jackson

**Affiliations:** 1 University of Otago, Wellington, New Zealand; 2 Te Aho o Te Kahu—Cancer Control Agency, Wellington, New Zealand; 3 Te Whatu Ora—Te Toka Tumai Auckland, Auckland, New Zealand; 4 Te Whatu Ora—Southern, Dunedin, New Zealand; 5 Te Whatu Ora—Counties Manukau, Auckland, New Zealand; 6 Te Whatu Ora—Waitematā, Auckland, New Zealand; 7 University of Waikato, Hamilton, New Zealand; 8 Department of Medicine, University of Otago, Dunedin, New Zealand

**Keywords:** Health services, Cancer therapy

## Abstract

**Background:**

Lung cancer survival can be improved through treatment, but several factors can influence the pattern of treatment received by a given individual. Using national-data across the lung cancer treatment pathway, here we describe treatment variation according to clinical and sociodemographic factors.

**Methods:**

We used national health data linked to the New Zealand Cancer Registry (NZCR) to describe variation in surgery, radiation therapy and systemic therapy for all lung cancers from 2012–2019 (*n* = 18,081), stratified by ethnicity, cancer stage, area deprivation, rurality status and comorbidity. Crude and adjusted descriptive analysis were used, including marginally standardised risk estimates from logistic regression.

**Results:**

The most common treatment was radiation therapy, either in isolation or combined with systemic therapy (combined total: crude 37%, fully-adj. 40%). Systemic therapy was the next most common treatment modality (crude 28%, fully-adj. 33%). Surgery was the least common treatment modality, used in around 15% of the cohort (crude 18%, fully-adj. 15%). Once adjusted for other factors, treatment access varied somewhat by ethnicity. Increasing stage of disease reduced the likelihood of surgery and increased the likelihood of radiation and systemic therapy. Increasing deprivation marginally reduced access to surgery, while rurality appeared to have no independent impact. Increasing comorbidity reduced the overall likelihood of treatment, systemic therapy alone, and radiation therapy + systemic therapy in combination.

**Discussion:**

We found variation in the pattern of cancer treatment received according to factors including ethnicity, stage, deprivation and comorbidity, but no clear independent variation by rurality.

## Introduction

Lung cancer is by far the number one cancer killer globally, responsible for 18% of all cancer deaths or around 1.8 million deaths each year [1]. While survival outcomes following a diagnosisof lung cancer are generally poor, advances in treatment have led to substantial improvements in lung cancer survival in recent years, particularly among those with advanced disease [2, 3].

There are multiple factors that influence the type of care that those diagnosed with lung cancer receive, which can be broadly categorised as clinical versus sociodemographic in nature. In terms of clinical considerations, the stage of disease at diagnosis will dictate whether curative or palliative treatment should be undertaken and also likely allow the application of a largely-standardised treatment pathway involving some combination of surgery, radiation therapy and/or systemic therapy [4]. The type of lung cancer (small-cell, non-small cell or other), histological sub-type of non-small cell lung cancer (NSCLC) (e.g. adenocarcinoma), and further molecular testing will also inform the most efficacious treatment pathway [4]. Even within a given stage of disease and tumour type, the extent of patient comorbidity will also influence clinical treatment decisions [5].

In terms of sociodemographic factors, there is evidence of a ‘post code lottery’ in lung cancer treatment, wherein those who live in rural areas may be less likely to access some forms of treatment compared to those living in urban treatment hubs [6]. There is some evidence that those living in socioeconomic deprivation are less likely to access best-practice lung cancer treatment for their stage of disease compared to those living in more affluent conditions [7]. Our own recent data also suggests that there are ethnic differences in access to lung cancer treatment, with these differences likely to contribute to known ethnic disparities in lung cancer survival [8].

The clinical reality is that none of the above factors occurs in a silo, but rather intersect and interact with each other. For example, looking beyond the disparities in access to the social determinants of good health that drive all ethnic disparities in access to cancer treatment [9], ethnic disparities in access to lung cancer treatment may be driven by differences in stage of disease at diagnosis, access to resources, rurality, comorbidity, or a combination of these. The complex nature of these interacting factors makes it difficult to assess the true nature of variation in lung cancer treatment by any one individual factor, which in turn limits our understanding of the extent to which this variation might be modifiable (and at what level an intervention might apply).

The purpose of this study was to describe how the pattern of lung cancer treatment varies according to these clinical and sociodemographic factors, adjusted for the confounding and/or mediating impact of the other factors that might influence this relationship. We have described this variation by sociodemographic factors including ethnicity, deprivation and rurality, as well as clinical factors including stage of disease and patient comorbidity at time of diagnosis.

## Methods

### Participants and data sources

The cohort included all lung cancer registrations (ICD-10-AM code: C33–C34) from 2012–2019 as listed on the New Zealand Cancer Registry (NZCR; *N* = 18,081). The NZCR is a compulsory record of all malignancies diagnosed in New Zealand except basal and squamous cell skin tumours [10]. The cohort identified from the NZCR were then linked via a unique, patient-specific and encrypted National Health Index number (NHI) to national healthcare collection data, including public and private hospital inpatient records (National Minimum Dataset, NMDS), the national Pharmaceutical Collection (PHARMS) dataset, and the Radiation Oncology Collection (ROC). Additional data sources including the Mortality Collection (death registrations), National Non-Admitted Patient Collection (NNPAC, outpatient and emergency department attendances) and Primary Health Organisation Enrolment Collection (primary care enrolments) were also linked to the NZCR cohort to supplement demographic information where NZCR records were incomplete or non-specific.

### Demographic variables

*Age at diagnosis* was derived from the NZCR, and categorised as <50, 50–64, 65–74, and 75+ years. *Prioritised ethnicity* was also derived from the NZCR (or supplementary sources as detailed above) and categorised into Māori, Pacific, Asian, European, or Other. *Area-level socioeconomic deprivation* was defined using the NZDep index of area deprivation [11], which determines area deprivation based on the domicile code available on the NZCR (or other supplementary sources as detailed above). NZDep is determined based on nine socio-economic deprivation variables available for just over 4 million people, and was calculated from 24,000 small areas containing a median of around 90 people in each area [11]. NZDep was categorised into five decile categories (NZDep 1–2 [least deprived], 3–4, 5–6, 7–8, 9–10 [most deprived]). *Rurality* was defined using the Geographical Classification for Health (GCH), based on domicile code [12]. The GCH categorises urban/rural status based on travel time to the edge of an urban area and population density, with categories including Urban 1 (‘most’ urban), Urban 2, Rural 1, Rural 2, Rural 3 (‘most’ rural) [12]. *Comorbidity* at cancer diagnosis was defined using the C3 cancer comorbidity index, which uses inpatient hospitalisation data from the 5-year period prior to cancer diagnosis to identify 42 individual conditions, after which a weighted score is attributed to each individual according to the conditions they have and the relationship of those conditions with non-cancer mortality in a cancer population [13]. C3 comorbidity score was categorised as ‘0’ (score ≤ 0), ‘1’ (>0, ≤1), ‘2’ (>1, ≤2) and ‘3’ (>2). If no recorded comorbidity was found over the 5-year period prior to diagnosis, individuals were assigned a score of 0 [13]. The C3 index was used as a categorical variable within descriptive analysis, and as a splined variable for logistic regression modelling/marginal standardisation using restricted cubic splines with three knots placed at the 75th, 90th and 95th percentiles [14].

### Tumour variables

*Tumour type* was defined using morphology data from the NZCR, and classified as small cell lung cancer (SCLC), NSCLC, or ‘other/unspecified’ [15]. *Stage of disease* was also derived from the NZCR, using the SEER Summary Stage method (A–F) [16], with stage collapsed for the purposes of this study into Local (SEER Summary Stage B), Regional (C and D), Advanced (E) and Unstaged (F) [17]. SEER stage was used due to the incomplete nature of TNM stage reporting on the NZCR.

### Treatment variables

*Receipt of surgery* was defined using public and private hospitalisation (NMDS) data, with procedures extracted using Australasian College of Health Informatics (ACHI) ICD-10-AM codes (2^nd^ Edition). Relevant curative and palliative surgical procedures were identified by our clinical advisors and are presented elsewhere [8]. We included all procedures that occurred between the period 90 days prior to cancer diagnosis, and up to 1 year post-diagnosis [8]. We used this pre-diagnosis period of 90 days to allow for the possibility that a relevant procedure occurred within this short period prior to a firm diagnosis and diagnosis date being set on the registry [8]. A binary indicator was set (yes/no) to indicate whether an individual underwent any of the included procedures within the treatment window.

*Receipt of radiation therapy* was derived from the ROC, and defined in a similar way to receipt of surgery. We scanned ROC data for instances of receipt of radiation treatment among our cohort during the treatment window (90 days prior to diagnosis, 1-year post-diagnosis), and set a binary indicator (yes/no) denoting whether someone received any radiation as part of their treatment.

*Receipt of systemic therapy* was derived from the PHARMS dataset using relevant chemical codes identified by our clinical advisors, with these codes listed elsewhere [8]. Like surgery and radiation therapy, this list of codes was used to scan the PHARMS data for receipt of systemic therapy (chemotherapy and funded targeted therapy, 90 days prior to diagnosis, up to 1 year after diagnosis) and a binary indicator (yes/no) was set to indicate whether someone underwent systemic therapy.

### Statistical analysis

#### Creation of treatment combination categories

After creating flags for each individual within our cohort according to whether they received surgery, radiation therapy and/or systemic therapy during our treatment window, we then created categories that identified which combination of treatment(s) were received during this window. These included a separate category for each possible combination of these three treatment types.

#### Descriptive analysis

Crude numbers and proportions (%) were determined for each possible treatment combination using standard descriptive analysis. Age-standardised proportions (%) were calculated through direct age standardisation using the *proc stdrate* procedure in SAS v9.4 (SAS Institute Inc., North Carolina, USA), with the total Māori lung cancer population as the standard population [18]. Within our results presentation, age-standardised proportions are labelled as ‘age adjusted %’.

#### Marginal standardisation

In order to adjust for the potential confounding and/or mediating impact of other factors on access to a given treatment combination, we conducted marginal standardisation based on logistic regression modelling [19] of the outcomes using the *margins* macro in SAS with the Māori lung cancer covariate profile as the reference. For example, the marginally-adjusted proportion of those with a C3 comorbidity score of >2 who only received surgery was adjusted for age, sex, ethnicity, deprivation, rurality, tumour type and stage, to adjust for the impact of these plausible confounding and mediating factors. The *rcspline* macro was used to include the C3 comorbidity score as a splined variable when used as a mediating factor in the marginal standardisation [20]. Within our results presentation, marginally-adjusted proportions are labelled as ‘fully adjusted %’.

#### Regression modelling

In addition to marginal standardisation, we explored relative differences between groups using logistic regression modelling (using the SAS procedure *proc logistic*) and determined odds ratios (ORs) adjusted for covariates (as per the marginal standardisation analysis).

Analysis was completed using SAS v9.4 (SAS Institute Inc., North Carolina, USA) and Microsoft Excel (Microsoft Corporation, Washington, USA).

## Results

The sociodemographic, tumour and comorbidity characteristics of the cohort are listed in Supplementary Material 1, with these data also presented separately by ethnic group in Supplementary Material 2. The pattern of treatment combinations observed for the total lung cancer cohort are shown in Supplementary Material 3. We found that the most common treatment received by those with lung cancer over our study period was radiation only (crude proportion of all those with lung cancer: 22%, marginally standardised to the Māori lung cancer covariate profile [‘fully-adj.’] 22%), followed by a combination of radiation plus systemic therapy (crude 15%, fully-adj. 18%). Around 15% of the cohort received some surgery, most commonly in isolation (surgery only crude 12%, full-adj. 9%; surgery plus radiation: crude 2%, fully-adj. 1%; surgery plus systemic therapy: crude/fully-adj. 3%; surgery plus radiation plus systemic therapy: crude 1%, fully-adj. 2%). Around 10% of the cohort received systemic therapy only (crude 9%, fully-adj. 10%).

### Variation by ethnicity

**Fig. 1:**
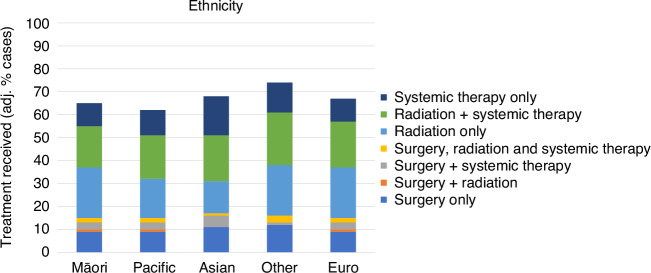
Variation in lung cancer treatment, by ethnicity. Proportions are adjusted for age, sex, area deprivation, rurality, comorbidity, stage and tumour type.

In general, there were few differences in the overall proportion of the cohort who received lung cancer treatment across ethnic groups (Fig. 1). In terms of combinations of that treatment, both Māori and European peoples appeared to receive similar combinations of lung cancer treatment. Pacific peoples appeared somewhat less likely to receive radiation only compared to the European population (crude 19%, fully-adj. 17%, adj. odds ratio [OR] 0.71, 95% CI 0.58–0.85), but had similar rates for all other treatment combinations – meaning that this deficit in radiation-only treatment was not explained by greater receipt of other treatment combinations. Asian peoples appeared more likely to undergo surgery than other ethnic groups (e.g. surgery only: crude 21%, crude OR 1.90, 95% CI 1.59–2.26), and this difference appeared to be substantially explained by differences between groups in one or more of the adjustment factors (fully adj. 11%, adj. OR 1.29, 95% CI 1.00–1.66), with Asian peoples less likely to have comorbidity and more likely to have earlier stage at diagnosis (Supplementary Material 2). Asian peoples with lung cancer were commensurately less likely to undergo radiation only (crude 15%), with this appearing to be uninfluenced by confounding and mediating factors (fully-adj. 14%, adj. OR 0.56, 95% CI 0.45–0.69). Data for the ‘Other’ group were sparse, preventing robust estimates of treatment combinations for this group (results not presented).

### Variation by stage

**Fig. 2:**
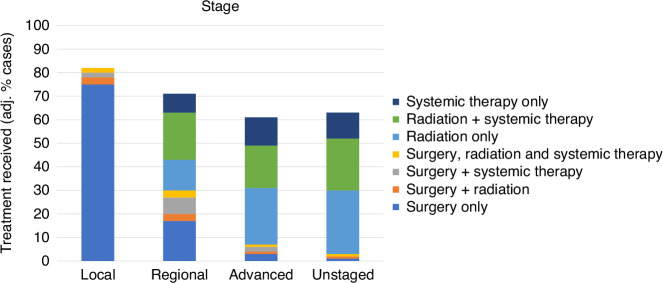
Variation in lung cancer treatment, by stage. Proportions are adjusted for age, sex, ethnicity, area deprivation, rurality, comorbidity and tumour type.

The overall proportion of the cohort who received treatment declined with increasing stage of disease, with the ‘Unstaged’ group receiving approximately the same volume of treatment as those with Advanced disease (Fig. 2). The primary form of treatment for those with Local disease was surgery, with nearly all patients undergoing surgery in some combination (combined totals: crude 95%, fully-adj. 82%), although surgery in isolation was the most common form (crude 86%, fully-adj. 75%). Receipt of surgery only reduced with increasing stage (e.g. surgery only, Regional stage: crude 23%, fully-adj. 17%; Advanced stage: crude 4%, fully adj. 3%), as did surgery plus radiation (Regional stage: crude 3%, fully-adj. 3%; Advanced stage: crude 1%, fully-adj. 1%) and surgery plus systemic therapy (Regional stage: crude 9%, fully-adj. 7%; Advanced stage: crude 2%, fully-adj. 2%). The rate of radiation only increased with increasing stage (Regional stage: crude 15%, fully-adj. 13%; Advanced stage: crude 24%, fully-adj. 24%), while radiation plus systemic therapy remained relatively static between those with Regional stage (crude 19%, fully-adj. 20%) and Advanced stage (crude 16%, fully-adj. 18%). Treatment combinations received by those with Unstaged disease somewhat mirrored that received by those with Advanced disease.

### Variation by deprivation

**Fig. 3:**
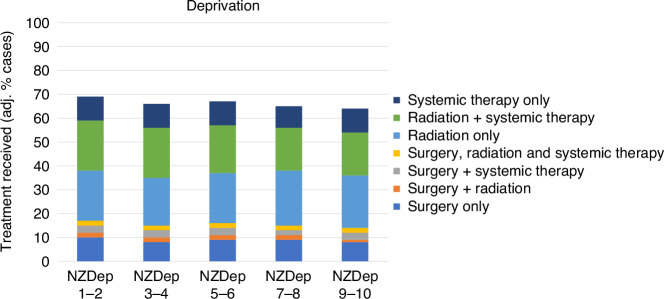
Variation in lung cancer treatment, by area deprivation. Proportions are adjusted for age, sex, ethnicity, rurality, comorbidity, stage and tumour type.

The overall proportion of the cohort who received treatment tended to reduce with increasing levels of deprivation, even after adjusting for all other covariates (Fig. 3). Deprivation did not appear to have an impact on the pattern of treatment combinations, with the possible exception of surgery, where those in the least deprived areas appeared to have marginally greater access to surgery than those in more deprived areas after adjusting for covariates (e.g. NZDep 1–2 [least deprived], surgery only: crude 15%, fully adj. 10%; NZDep 9–10 [most deprived]: crude 10%, fully adj. 8%; adj. OR 0.77, 95% CI 0.62-0.95).

### Variation by rurality

**Fig. 4:**
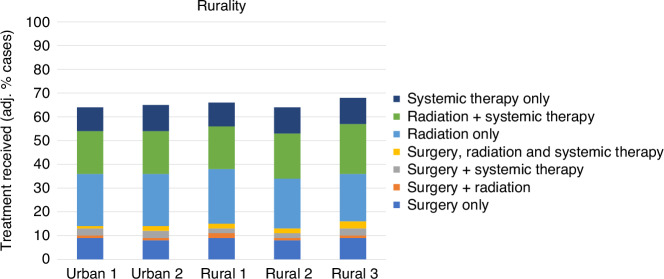
Variation in lung cancer treatment, by rurality. Proportions are adjusted for age, sex, ethnicity, area deprivation, comorbidity, stage and tumour type.

The overall proportion of the cohort who received treatment was similar across urban/rural categories (Fig. 4). There appeared to be no meaningful differences across urban/rural categories in terms of the pattern of treatment combinations, both within the crude results and the fully-adjusted results.

### Variation by comorbidity

**Fig. 5:**
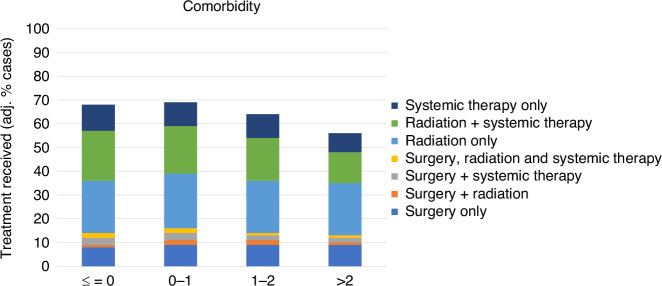
Variation in lung cancer treatment, by comorbidity. Proportions are adjusted for age, sex, ethnicity, area deprivation, rurality, stage and tumour type.

The overall proportion of the cohort who received treatment reduced with increasing level of comorbidity, although there did not appear to be a difference in this proportion between those with no comorbidity (C3 Index score <=0) versus some comorbidity (C3 Index score 0-1; Fig. 5). In terms of the pattern of treatment combinations, it appears this attenuation in treatment with increasing level of comorbidity is primarily due to a drop-off in the rate of systemic therapy (e.g. C3 Index ≤ 0: crude 10%, fully adj. 11%; C3 Index >2: crude 5%, fully adj. 8%; adj. OR 0.68, 95% CI 0.58–0.79) or radiation therapy plus systemic therapy (e.g. C3 Index ≤ 0: crude 19%, fully adj. 21%; C3 Index >2: crude 7%, fully adj. 13%; adj. OR 0.49, 95% CI 0.43–0.57).

## Discussion

Over our study period, the most common treatment received by those with lung cancer in Aotearoa New Zealand was radiation therapy, either in isolation or combined with systemic therapy (combined total: crude 37%). This trend held when adjusting for multiple factors, including stage of disease and comorbidity (fully-adj. 40%). Systemic therapy was the next most common treatment modality, used in around a third of the cohort (crude 28%, fully-adj. 33%). Surgery was the least common treatment modality, used in around 15% of the cohort (crude 18%, fully-adj. 15%).

Our observations regarding variation in treatment patterns by population sub-group are detailed below.

### Ethnicity

We observed that, once adjusted for all other covariates, Māori did not appear to be receiving a different pattern of lung cancer treatment compared to Europeans. At a glance, this suggests that the combination of treatment received by Māori with lung cancer is unlikely to be a contributor to enduring disparities in cancer survival for Māori. However, this finding must be considered alongside both our recent observation of lower surgical rates among Māori with lung cancer, where Māori were less likely to receive surgery (age-standardised %: Māori 14%, European 20%) including curative surgery (Māori 10%, European 16%) [8], as well as our observation in the current study that Māori were less likely to access surgery only before adjusting for covariates other than age (age-standardised %, combined surgery totals: Māori 15%, European 21%). In combination, these findings suggest that Māori are indeed less likely to receive surgery than Europeans, but that this difference is largely explained by differences between these groups in factors such as comorbidity and stage (Supplementary Material 2). The findings of the current study suggest that once these factors are accounted for, Māori receive largely the same pattern of lung cancer care as Europeans; but this should not be misinterpreted as meaning that there is no difference between Māori and Europeans in terms of received treatment. Our findings merely suggest that it there is no apparent impact of Māori ethnicity on pattern of care independent of factors such as stage and comorbidity.

We found that Pacific peoples appeared to be marginally less likely to access radiation therapy than other ethnic groups, with no commensurate increase in receipt of other forms of treatment—even after adjusting for plausible drivers of this deficit in treatment, including comorbidity and stage of disease. This appeared to be the primary contributor to the overall slightly lower rates of lung cancer treatment among Pacific peoples in the adjusted results. This slightly-reduced access to radiation therapy will not be related to geographic access to care, because we have already shown that Pacific peoples with lung cancer almost exclusively live within urban hubs with radiation treatment facilities [21]. While further work regarding this observation is warranted, we note that relatively low numbers of Pacific patients means that these findings may be imprecise, and therefore any differences should be interpreted with caution.

We noted that Asian peoples appear to be accessing more lung cancer surgery and less radiation therapy than other ethnic groups, which is partly explained by a tendency toward earlier stage of disease at diagnosis and less comorbidity among this cohort. A greater proportion of Asian patients with lung cancer are non-smokers [22]: in New Zealand, a previous study found that 35% of Asian patients with lung cancer were non-smokers compared to 8% of European patients and 1% of Māori patients [23]. This may lead to an increased propensity toward low-grade adenocarcinomas among Asian patients that should be amenable to surgery, and the patients were relatively young and fit enough to receive this treatment (around 70% of Asian patients in our cohort had a comorbidity score of ≤0; Supplementary Table 1).

### Stage

In terms of stage of disease, we found that surgery receipt goes down with increasing stage of disease, while the relative importance of systemic therapy and radiation therapy increases with increasing stage. Improving access to early detection of lung cancer through interventions including screening [24] would change this pattern by increasing the viability of surgery as a curative treatment option.

We observed an unexpected near-absence of radiation therapy only among those with Local stage. We expected to see more instances of stereotactic ablation radiation therapy (SABR) among this cohort, as SABR became a common treatment for early-stage lung cancer over our study period [25]. Within our own cohort, we found that use of SABR occurred in <1% of those with Local, Regional or Advanced disease, and 4% of those with Unstaged disease (crude %; data not shown). Recent data from a large clinical registry found that SABR was used to treat around 11% of early-stage lung cancer (TNM Stage I–II) cancers [26], reinforcing the unusually low proportion treated with SABR in the current study. The first plausible reason for this observation is that SABR was under-used in the New Zealand context over this study period, although there is limited evidence to suggest that this was the case [25]. A second plausible reason is that patients who receive SABR might not be registered on the NZCR, because the primary source of data for this registry is pathology records [10], and early-stage patients with lung cancer who receive SABR may be less likely to have a pathology sample taken as part of their care. A recent granular analysis from the same clinical registry found that a small proportion (6%) of their clinical cohort were not registered on the NZCR, and that around a third (29%) of this group had Stage I disease [27]. This observation, in addition to the near-absence of radiation therapy among those with early disease in our cohort, suggests (a) that we are likely missing a small proportion of cases from our cohort, (b) that this absence is not likely to be random but rather patterned by stage, with many of these missing registrations likely to be of Local stage, and (c) this absence precludes us from fully describing the pattern of treatment received by those with Local disease.

### Rurality

Contrary to expectations, rurality appears to have no impact on the combination of treatment received by lung cancer patients in Aotearoa, both before and after adjustment for possible confounding and mediating factors such as deprivation. This suggests that those living in rural areas are accessing the same patterns of care as those living in urban centres, despite having to overcome barriers to accessing surgery and radiation therapy which are almost exclusively delivered in our major cities [28]. This observation does not negate the need for us to decentralise care and provide it close to where people live wherever possible—particularly since treatment centralisation tends to make access easier for some population groups over others (e.g. odds ratio of Māori with lung cancer living ≥200 km away from radiation therapy: 1.41, 95% CI 1.25–1.60) [21].

### Deprivation

Increasing deprivation appeared to slightly reduce both the overall proportion of the cohort who received treatment as well as the proportion who received surgery only. While the difference in surgery rates was greatest prior to marginal standardisation for other covariates, a small disparity did remain even after adjustment for these factors. This reinforces the likelihood that deprivation can act as a barrier to treatment access independent of factors such as rurality, as well as the importance of considering the independent impact of deprivation on outcomes within equity-focussed analysis (e.g. ethnicity comparisons).

### Comorbidity

The overall proportion of treatment reduces with increasing comorbidity, with this deficit apparently driven by a reduction in receipt of systemic therapy (but not other treatments) as comorbidity increases. On the one hand, this observation is surprising. Previous research (including our own) has shown that the presence of comorbidity reduces the likelihood that a person with cancer will receive surgery; it appears that this impact does not apply to the lung cancer cohort, which may be explained by the heightened presence of comorbidity among those with lung cancer relative to other cancers [29]. (In other words, much of our lung cohort have comorbidity, so there is less opportunity for observing a strong relationship with treatment receipt.) On the other hand, despite the heightened morbidity within the cohort, we still observed a strong relationship between increasing comorbidity burden and likelihood of receiving systemic therapy only (C3 category ≤0 vs >2, adj. 11% vs. 8% respectively, adj. OR 0.68, 95% CI 0.58–0.79) and radiation therapy plus systemic therapy (21% vs. 13%, adj. OR 0.49, 0.43–0.57). In order to assess the extent to which the latter was driven primarily by systemic therapy access (as indicated by the systemic therapy only finding), or whether radiation therapy access was also a factor, we examined the receipt of any radiation therapy (regardless of combination with other treatment) by comorbidity status, and found that those with a C3 score of >2 were less likely to access any radiation therapy compared to those with a C3 score of ≤0 (adj. 46% vs. adj. 38%, adj. OR 0.71, 95% CI 0.65–0.78), despite there being no difference between these comorbidity groups in terms of access to radiation therapy only within the current study (both 22%). In combination, these findings suggest that comorbidity is influencing both the receipt of radiation therapy and systemic therapy, rather than surgery, independently from other factors including stage of disease (to the extent to which we can adjust for this factor with our current data). Further nuanced investigation of this trend with clinical registry data (and complete TNM staging and ECOG performance status data) will add granularity to this observation; however, in the meantime, our findings suggest that comorbidity is an important determinant of access to radiation therapy and systemic therapy in the lung cancer context, and as such the management of comorbidity among this population could improve access to this treatment, with subsequent improvement in outcomes.

### Recommendations

Based on the findings outlined above, we make the following recommendations regarding system- and clinical-level changes arising from this study:

- Patterns of treatment depend on stage of disease, and as such, a population shift toward early-detection of lung cancer would result in a corresponding shift away from the patterns of treatment observed for late stage disease (proportionally more systemic therapy) to those observed for early stage disease (proportionally more surgery). A lung cancer screening programme is one means by which such a shift could occur [24].
- Comorbidity independently influences access to radiation therapy and systemic therapy, and therefore, the primary prevention of morbidities including diabetes, heart disease and others would serve to improve access to these cancer therapies among those with lung cancer. In addition, management of comorbidity and prehabilitation activities could also improve treatment access—with such an improvement set to disproportionately improve access for those populations with an inequitable burden of comorbidity (such as Māori and Pacific).
- Pacific peoples appear to not be accessing radiation therapy to the same extent as other ethnic groups, even after adjusting for factors including comorbidity. Further work is required to understand the potential barriers that Pacific peoples may uniquely face in accessing this care.
- Rurality does not seem to influence either the pattern of lung cancer treatment received or the overall likelihood of treatment—but that does not mean that our rural populations are not experiencing barriers to care. Rather, it suggests that these barriers are being overcome by a resilient rural population, but since much of lung cancer treatment is delivered in central hubs [21], this resilience will come at a price. Any and all activities that aim to reduce rural barriers to cancer care access, including improvement in the availability of up-front travel expense funding through transport assistance programmes, are sorely needed in acknowledgement of the systematic problem that centralisation of treatment creates for those diagnosed with cancer while living in rural communities.

### Strengths and limitations

A key strength of this study is the use of national-level data, maximising the generalisability of our findings to the lung cancer population within New Zealand. As noted above, it is likely that we are missing a small number of the total incident lung cancers in New Zealand over the study period, and while this likely undercounts receipt of radiation therapy among our cohort (as outlined above), this should not meaningfully impact the key themes of our observations. We noted above the limitations of the staging information currently available at a national level for lung cancer in New Zealand; this limits the granularity of our observations, and reinforces the importance of rapid improvement in the availability and linkage of clinical staging to national health records, with work currently underway in New Zealand to address this gap [30]. Finally, we note that since our study cohort ceased at the end of 2019, there may have been some changes to treatment modality since this time, such as increased use of immunotherapy and targeted therapy in the treatment of more advanced disease.

## Conclusions

In this study we found variation in the pattern of cancer treatment received according to factors including ethnicity, stage, deprivation and comorbidity, but no clear variation in treatment pattern by rurality. This absence of variation by rurality in the context of a cancer where care is primarily delivered in central hubs is striking, but does not mitigate the need for continual improvement in support for rural patients accessing care, as well as decentralisation of services where possible. Stage, comorbidity and deprivation remain key factors that drive access to curative treatment and subsequent outcomes. Activities that aim to shift the distribution of stage at diagnosis to earlier in the disease pathway, prevent or manage comorbidity, and make treatment access as well-supported and straightforward as possible for those living in deprivation or in rural areas, will collectively serve to improve access to lung cancer treatment and subsequent outcomes.

## Supplementary information

Supplementary Material

## Data Availability

Data cannot be shared publicly because of data restrictions put in place by the New Zealand Government. The data underlying the results presented in this study are available from the National Collections team at Te Whatu Ora—Health New Zealand, following a project review and approval process. For further information regarding access to these data, email data-enquiries@health.govt.nz.
