# Supplementary material for: National variation in the treatment of lung cancer in a universal healthcare context
Source: Br J Cancer. 2025 Jun 6;133(3):346–52. doi: 10.1038/s41416-025-03071-9 (PMC12322026; doi:10.1038/s41416-025-03071-9)
Supplement: Supplementary file 1 — Supplementary Material [file 41416_2025_3071_MOESM1_ESM.docx]

# Supplementary Material

- Supplementary Material 1: Characteristics of the cohort (lung cancers on NZCR, 2012-2019).
- Supplementary Material 2: Characteristics of the cohort (lung cancers on NZCR, 2012-2019), stratified by ethnicity.
- Supplementary Material 3: Lung cancer treatment for the total cohort, including crude, age-standardised and marginally-standardised results.
- Supplementary Material 4: Variation in lung cancer treatment by ethnicity, including crude, age-standardised and marginally-standardised results.
- Supplementary Material 5: Variation in lung cancer treatment by stage, including crude, age-standardised and marginally-standardised results.
- Supplementary Material 6: Variation in lung cancer treatment by deprivation, including crude, age-standardised and marginally-standardised results.
- Supplementary Material 7: Variation in lung cancer treatment by rurality, including crude, age-standardised and marginally-standardised results.
- Supplementary Material 8: Variation in lung cancer treatment by comorbidity, including crude, age-standardised and marginally-standardised results.

**Supplementary Material 1:** Characteristics of the cohort (lung cancers on NZCR, 2012-2019).

|  | **Total** | | |  |
| --- | --- | --- | --- | --- |
|  | *N* | *Crude %* | |  |
| **Total** | 18,081 | - | |  |
| **Sex** |  |  | |  |
| Female | 8,926 | 49% | |  |
| Male | 9,155 | 51% | |  |
| **Age** (years) |  |  | |  |
| <50 | 673 | 4% | |  |
| 50-64 | 4,526 | 25% | |  |
| 65-74 | 6,106 | 34% | |  |
| 75+ | 6,776 | 37% | |  |
| **Ethnicity** |  |  | |  |
| Māori | 3,762 | 21% | |  |
| Pacific | 857 | 5% | |  |
| Asian | 839 | 5% | |  |
| MELAA/Other | 88 | 0% | |  |
| European | 12,535 | 69% | |  |
| **Deprivation** (NZDep decile) |  |  | |  |
| 1-2 (least deprived) | 2,278 | 13% | |  |
| 3-4 | 2,598 | 14% | |  |
| 5-6 | 3,467 | 19% | |  |
| 7-8 | 4,416 | 24% | |  |
| 9-10 (most deprived) | 5,275 | 29% | |  |
| Missing | 47 |  | |  |
| **Rurality** (GCH Category) |  |  | |  |
| Urban 1 | 9,677 | 54% | |  |
| Urban 2 | 3,965 | 22% | |  |
| Rural 1 | 2,769 | 15% | |  |
| Rural 2 | 1,364 | 8% | |  |
| Rural 3 | 260 | 1% | |  |
| Missing | 46 |  | |  |
| **Comorbidity** (C3 Index score) | | |  | |
| <=0 | 8,667 | 48% | |  |
| 0-1 | 2,505 | 14% | |  |
| 1-2 | 2,693 | 15% | |  |
| >2 | 4,216 | 23% | |  |
| **Tumour Type** |  |  | |  |
| Small Cell | 1,916 | 11% | |  |
| Non-Small Cell | 11,925 | 66% | |  |
| Other/Unspecified | 4,240 | 23% | |  |
| **Stage** |  |  | |  |
| Local | 1,244 | 7% | |  |
| Regional | 2,750 | 15% | |  |
| Advanced | 8,087 | 45% | |  |
| Unstaged | 6,000 | 33% | |  |
|  |  |  | |  |

**Supplementary Material 2:** Characteristics of the cohort (lung cancers on NZCR, 2012-2019), stratified by ethnicity.

|  | **Māori** | | | **Pacific** | | | **Asian** | | | **MELAA/Other** | | | **European** | | |
| --- | --- | --- | --- | --- | --- | --- | --- | --- | --- | --- | --- | --- | --- | --- | --- |
|  | *n* | *Crude %* | *Age Std. %* | *n* | *Crude %* | *Age Std. %* | *n* | *Crude %* | *Age Std. %* | *n* | *Crude %* | *Age Std. %* | *n* | *Crude %* | *Age Std. %* |
| **Total** | 3,762 | 21% | 25% | 857 | 5% | 5% | 839 | 5% | 5% | 88 | 0% | 1% | 12,535 | 69% | 64% |
| **Sex** |  |  |  |  |  |  |  |  |  |  |  |  |  |  |  |
| Female | 2,133 | 57% | 57% | 330 | 39% | 38% | 402 | 48% | 47% | 32 | 36% | 33% | 6,029 | 48% | 50% |
| Male | 1,629 | 43% | 43% | 527 | 61% | 62% | 437 | 52% | 53% | 56 | 64% | 67% | 6,506 | 52% | 50% |
| **Age** (years) |  |  |  |  |  |  |  |  |  |  |  |  |  |  |  |
| <50 | 201 | 5% |  | 67 | 8% |  | 86 | 10% |  | 7 | 8% |  | 312 | 2% |  |
| 50-64 | 1,534 | 41% |  | 285 | 33% |  | 249 | 30% |  | 29 | 33% |  | 2,429 | 19% |  |
| 65-74 | 1,298 | 35% |  | 271 | 32% |  | 261 | 31% |  | 20 | 23% |  | 4,256 | 34% |  |
| 75+ | 729 | 19% |  | 234 | 27% |  | 243 | 29% |  | 32 | 36% |  | 5,538 | 44% |  |
| **Deprivation** (NZDep decile) |  |  |  |  |  |  |  |  |  |  |  |  |  |  |  |
| 1-2 (least deprived) | 134 | 4% | 4% | 34 | 4% | 4% | 182 | 22% | 23% | 17 | 19% | 16% | 1,911 | 15% | 16% |
| 3-4 | 288 | 8% | 8% | 68 | 8% | 8% | 181 | 22% | 21% | 17 | 19% | 18% | 2,044 | 16% | 16% |
| 5-6 | 454 | 12% | 12% | 87 | 10% | 10% | 190 | 23% | 23% | 16 | 18% | 19% | 2,720 | 22% | 21% |
| 7-8 | 869 | 23% | 23% | 161 | 19% | 19% | 146 | 17% | 17% | 21 | 24% | 23% | 3,219 | 26% | 25% |
| 9-10 (most deprived) | 2,016 | 54% | 54% | 494 | 58% | 58% | 137 | 16% | 16% | 15 | 17% | 21% | 2,613 | 21% | 21% |
| Missing | 1 | 0% | 0% | 13 | 2% | 2% | 3 | 0% | 0% | 2 | 2% | 2% | 28 | 0% | 0% |
| **Rurality** (GCH Category) |  |  |  |  |  |  |  |  |  |  |  |  |  |  |  |
| Urban 1 | 1,608 | 43% | 43% | 743 | 87% | 87% | 769 | 92% | 91% | 68 | 77% | 80% | 6,489 | 52% | 51% |
| Urban 2 | 968 | 26% | 26% | 50 | 6% | 6% | 34 | 4% | 4% | 10 | 11% | 7% | 2,903 | 23% | 23% |
| Rural 1 | 601 | 16% | 16% | 35 | 4% | 4% | 29 | 3% | 3% | 6 | 7% | 8% | 2,098 | 17% | 17% |
| Rural 2 | 453 | 12% | 12% | 14 | 2% | 2% | 3 | 0% | 0% | 2 | 2% | 2% | 892 | 7% | 7% |
| Rural 3 | 131 | 3% | 3% | 2 | 0% | 0% | 1 | 0% | 0% | - | 0% | 0% | 126 | 1% | 1% |
| Missing | 1 | 0% | 0% | 13 | 2% | 2% | 3 | 0% | 0% | 2 | 2% | 2% | 27 | 0% | 0% |
| **Comorbidity** (C3 Index score) |  |  |  |  |  |  |  |  |  |  |  |  |  |  |  |
| <=0 | 1,717 | 46% | 46% | 436 | 51% | 52% | 557 | 66% | 68% | 52 | 59% | 58% | 5,905 | 47% | 54% |
| 0-1 | 457 | 12% | 12% | 83 | 10% | 10% | 92 | 11% | 11% | 12 | 14% | 14% | 1,861 | 15% | 14% |
| 1-2 | 602 | 16% | 16% | 122 | 14% | 14% | 85 | 10% | 10% | 12 | 14% | 14% | 1,872 | 15% | 14% |
| >2 | 986 | 26% | 26% | 216 | 25% | 24% | 105 | 13% | 11% | 12 | 14% | 13% | 2,897 | 23% | 18% |
| **Tumour Type** |  |  |  |  |  |  |  |  |  |  |  |  |  |  |  |
| Small Cell | 610 | 16% | 16% | 81 | 9% | 10% | 45 | 5% | 6% | 4 | 5% | 4% | 1,176 | 9% | 11% |
| Non-Small Cell | 2,403 | 64% | 64% | 630 | 74% | 76% | 698 | 83% | 84% | 66 | 75% | 77% | 8,128 | 65% | 70% |
| Other/Unspecified | 749 | 20% | 20% | 146 | 17% | 14% | 96 | 11% | 10% | 18 | 20% | 19% | 3,231 | 26% | 19% |
| **Stage** |  |  |  |  |  |  |  |  |  |  |  |  |  |  |  |
| Local | 163 | 4% | 4% | 49 | 6% | 6% | 109 | 13% | 14% | 6 | 7% | 6% | 917 | 7% | 9% |
| Regional | 628 | 17% | 17% | 122 | 14% | 15% | 162 | 19% | 20% | 18 | 20% | 24% | 1,820 | 15% | 16% |
| Advanced | 1,695 | 45% | 45% | 476 | 56% | 55% | 377 | 45% | 46% | 38 | 43% | 43% | 5,501 | 44% | 46% |
| Unstaged | 1,276 | 34% | 34% | 210 | 25% | 23% | 191 | 23% | 21% | 26 | 30% | 27% | 4,297 | 34% | 29% |
|  |  |  |  |  |  |  |  |  |  |  |  |  |  |  |  |

**Supplementary Material 3:** Lung cancer treatment for the total cohort, including crude, age-standardised and marginally-standardised results.

|  | *n* | *Crude %* | *Age Adj. %* | *Fully Adj. %* |
| --- | --- | --- | --- | --- |
| **Total Cohort** |  |  |  |  |
| Surgery Only | 2,154 | 12% | 13% | 9% |
| Surgery + Radiation | 285 | 2% | 2% | 1% |
| Surgery + Systemic Therapy | 486 | 3% | 3% | 3% |
| Surgery, Radiation and Systemic Therapy | 256 | 1% | 2% | 2% |
|  |  |  |  |  |
| Radiation Only | 4,042 | 22% | 21% | 22% |
| Radiation + Systemic Therapy | 2,629 | 15% | 18% | 18% |
|  |  |  |  |  |
| Systemic Therapy Only | 1,553 | 9% | 10% | 10% |
|  |  |  |  |  |

**Supplementary Material 4:** Variation in lung cancer treatment by ethnicity, including crude, age-standardised and marginally-standardised results.

|  |  |  |  |  | Odds Ratios (95% CI) | |
| --- | --- | --- | --- | --- | --- | --- |
|  | n | % | Age Adj. % | Fully Adj. % | Crude OR | Adj. OR |
| Māori |  |  |  |  |  |  |
| Surgery Only | 321 | 9% | 9% | 9% | 0.66 (0.58-0.75) | 0.83 (0.70-0.99) |
| Surgery + Radiation | 52 | 1% | 1% | 1% | 0.81* (0.59-1.09) | 1.03 (0.74-1.44) |
| Surgery + Systemic Therapy | 99 | 3% | 3% | 3% | 1.09 (0.87-1.38) | 0.87 (0.67-1.13) |
| Surgery, Radiation and Systemic Therapy | 60 | 2% | 2% | 2% | 1.25 (0.93-1.69) | 0.89 (0.64-1.24) |
|  |  |  |  |  |  |  |
| Radiation Only | 828 | 22% | 22% | 22% | 0.93 (0.85-1.02) | 1.00 (0.90-1.10) |
| Radiation + Systemic Therapy | 692 | 18% | 18% | 18% | 1.49 (1.35-1.64) | 0.90 (0.80-1.02) |
|  |  |  |  |  |  |  |
| Systemic Therapy Only | 384 | 10% | 10% | 10% | 1.37 (1.21-1.55) | 1.06 (0.92-1.22) |
|  |  |  |  |  |  |  |
| Pacific |  |  |  |  |  |  |
| Surgery Only | 87 | 10% | 11% | 9% | 0.80 (0.64-1.01) | 0.85 (0.62-1.16) |
| Surgery + Radiation | 14 | 2% | 2% | 1% | 0.97* (0.56-1.67) | 1.04 (0.59-1.85) |
| Surgery + Systemic Therapy | 27 | 3% | 3% | 3% | 1.34 (0.90-1.99) | 0.93 (0.60-1.43) |
| Surgery, Radiation and Systemic Therapy | 19 | 2% | 2% | 2% | 1.78 (1.10-2.87) | 1.31 (0.78-2.20) |
|  |  |  |  |  |  |  |
| Radiation Only | 161 | 19% | 19% | 17% | 0.78 (0.65-0.93) | 0.71 (0.58-0.85) |
| Radiation + Systemic Therapy | 133 | 16% | 16% | 19% | 1.23 (1.02-1.49) | 0.94 (0.75-1.17) |
|  |  |  |  |  |  |  |
| Systemic Therapy Only | 82 | 10% | 10% | 11% | 1.29 (1.02-1.64) | 1.18 (0.91-1.52) |
|  |  |  |  |  |  |  |
| Asian |  |  |  |  |  |  |
| Surgery Only | 178 | 21% | 22% | 11% | 1.90 (1.59-2.26) | 1.29 (1.00-1.66) |
| Surgery + Radiation | 5 | 1% | - | - | 0.35* (0.14-0.84) | 0.23 (0.09-0.57) |
| Surgery + Systemic Therapy | 57 | 7% | 7% | 5% | 2.96 (2.21-3.96) | 1.64 (1.20-2.25) |
| Surgery, Radiation and Systemic Therapy | 14 | 2% | 2% | 1% | 1.31 (0.76-2.28) | 0.82 (0.47-1.46) |
|  |  |  |  |  |  |  |
| Radiation Only | 123 | 15% | 14% | 14% | 0.57 (0.47-0.69) | 0.56 (0.45-0.69) |
| Radiation + Systemic Therapy | 138 | 16% | 18% | 20% | 1.30 (1.08-1.57) | 1.01 (0.82-1.25) |
|  |  |  |  |  |  |  |
| Systemic Therapy Only | 117 | 14% | 14% | 17% | 1.96 (1.59-2.41) | 1.98 (1.59-2.47) |
|  |  |  |  |  |  |  |
| MELAA/Other |  |  |  |  |  |  |
| Surgery Only | 16 | 18% | 17% | 12% | 1.49 (0.85-2.61) | 1.50 (0.73-3.06) |
| Surgery + Radiation | 0 | 0% | - | - | - | - |
| Surgery + Systemic Therapy | 1 | 1% | - | - | - | - |
| Surgery, Radiation and Systemic Therapy | 3 | 3% | - | - | 2.79 (0.87-8.92) | 1.90 (0.58-6.24) |
|  |  |  |  |  |  |  |
| Radiation Only | 21 | 24% | 21% | 22% | 1.07 (0.65-1.75) | 1.03 (0.62-1.73) |
| Radiation + Systemic Therapy | 16 | 18% | 20% | 23% | 1.51 (0.87-2.60) | 1.29 (0.70-2.35) |
|  |  |  |  |  |  |  |
| Systemic Therapy Only | 9 | 10% | 12% | 13% | 1.41 (0.70-2.81) | 1.49 (0.73-3.05) |
|  |  |  |  |  |  |  |
| European |  |  |  |  |  |  |
| Surgery Only | 1552 | 12% | 14% | 9% | Ref | Ref |
| Surgery + Radiation | 214 | 2% | 2% | 1% | Ref | Ref |
| Surgery + Systemic Therapy | 302 | 2% | 3% | 3% | Ref | Ref |
| Surgery, Radiation and Systemic Therapy | 160 | 1% | 2% | 2% | Ref | Ref |
|  |  |  |  |  |  |  |
| Radiation Only | 2909 | 23% | 22% | 22% | Ref | Ref |
| Radiation + Systemic Therapy | 1650 | 13% | 19% | 20% | Ref | Ref |
|  |  |  |  |  |  |  |
| Systemic Therapy Only | 961 | 8% | 9% | 10% | Ref | Ref |
|  |  |  |  |  |  |  |

**Note:** Age adj. %: age-standardised; Fully adj. % = marginally-standardised. Adj. OR: adjusted for age, sex, deprivation, rurality, comorbidity, tumour type and stage. Dash (-) indicates that data are supressed due to insufficient data (~<10 cases within the strata). Asterisk (*) indicates caution when interpreting data due to a lack of model convergence. Age standardised and fully standardised rates not produced where count is 5 or fewer.

**Supplementary Material 5:** Variation in lung cancer treatment by stage, including crude, age-standardised and marginally-standardised results.

|  |  |  |  |  | Odds Ratios (95% CI) | | |
| --- | --- | --- | --- | --- | --- | --- | --- |
|  | n | % | Age Adj. % | Fully Adj. % | Crude OR | Adj. OR |  |
| **Local** |  |  |  |  |  |  |  |
| Surgery Only | 1075 | 86% | 86% | 75% | Ref | Ref |  |
| Surgery + Radiation | 51 | 4% | 4% | 3% | Ref | Ref |  |
| Surgery + Systemic Therapy | 43 | 3% | 4% | 2% | Ref | Ref |  |
| Surgery, Radiation and Systemic Therapy | 21 | 2% | 2% | 2% | Ref | Ref |  |
|  |  |  |  |  |  |  |  |
| Radiation Only | 1 | 0% | - | - | Ref | Ref |  |
| Radiation + Systemic Therapy | 0 | 0% | - | - | - | - |  |
|  |  |  |  |  |  |  |  |
| Systemic Therapy Only | 3 | 0% | - | - | Ref | Ref |  |
|  |  |  |  |  |  |  |  |
| **Regional** |  |  |  |  |  |  |  |
| Surgery Only | 626 | 23% | 22% | 17% | 0.05 (0.04-0.06) | 0.05 (0.04-0.06) |  |
| Surgery + Radiation | 95 | 3% | 3% | 3% | 0.84 (0.59-1.19) | 0.90 (0.63-1.28) |  |
| Surgery + Systemic Therapy | 242 | 9% | 10% | 7% | 2.70 (1.94-3.76) | 3.28 (2.34-4.60) |  |
| Surgery, Radiation and Systemic Therapy | 96 | 3% | 4% | 3% | 2.11 (1.31-3.40) | 2.18 (1.35-3.54) |  |
|  |  |  |  |  |  |  |  |
| Radiation Only | 422 | 15% | 15% | 13% | 224.90 (31.58-1601.60) | 241.16 (33.86-1717.50) |  |
| Radiation + Systemic Therapy | 516 | 19% | 21% | 20% | - | - |  |
|  |  |  |  |  |  |  |  |
| Systemic Therapy Only | 216 | 8% | 8% | 8% | 35.11 (11.21-109.91) | 32.63 (10.42-102.25) |  |
|  |  |  |  |  |  |  |  |
| **Advanced** |  |  |  |  |  |  |  |
| Surgery Only | 339 | 4% | 4% | 3% | 0.01 (0.01-0.01) | 0.01 (0.01-0.01) |  |
| Surgery + Radiation | 108 | 1% | 1% | 1% | 0.32 (0.23-0.45) | 0.39 (0.28-0.56) |  |
| Surgery + Systemic Therapy | 181 | 2% | 3% | 2% | 0.64 (0.46-0.90) | 0.87 (0.62-1.23) |  |
| Surgery, Radiation and Systemic Therapy | 108 | 1% | 2% | 1% | 0.79 (0.49-1.27) | 0.88 (0.54-1.41) |  |
|  |  |  |  |  |  |  |  |
| Radiation Only | 1948 | 24% | 24% | 24% | 395.54 (55.66-2810.90) | 512.88 (72.16-3645.10) |  |
| Radiation + Systemic Therapy | 1313 | 16% | 20% | 18% | - | - |  |
|  |  |  |  |  |  |  |  |
| Systemic Therapy Only | 873 | 11% | 12% | 12% | 50.22 (16.15-156.23) | 51.06 (16.40-159.01) |  |
|  |  |  |  |  |  |  |  |
| **Unstaged** |  |  |  |  |  |  |  |
| Surgery Only | 114 | 2% | 2% | 1% | 0.00 (0.00-0.00) | 0.00 (0.00-0.00) |  |
| Surgery + Radiation | 31 | 1% | 1% | 1% | 0.12 (0.08-0.19) | 0.16 (0.10-0.26) |  |
| Surgery + Systemic Therapy | 20 | 0% | 1% | 0% | 0.09 (0.05-0.16) | 0.18 (0.10-0.30) |  |
| Surgery, Radiation and Systemic Therapy | 31 | 1% | 1% | 1% | 0.30 (0.17-0.53) | 0.48 (0.28-0.85) |  |
|  |  |  |  |  |  |  |  |
| Radiation Only | 1671 | 28% | 27% | 27% | 480.10 (67.55-3412.39) | 617.26 (86.83-4388.18) |  |
| Radiation + Systemic Therapy | 800 | 13% | 21% | 22% | - | - |  |
|  |  |  |  |  |  |  |  |
| Systemic Therapy Only | 461 | 8% | 10% | 11% | 34.43 (11.05-107.31) | 47.29 (15.15-147.55) |  |
|  |  |  |  |  |  |  |  |

**Note:** Age adj. %: age-standardised; Fully adj. % = marginally-standardised. Adj. OR: adjusted for age, sex, deprivation, ethnicity, rurality, comorbidity and tumour type. Dash (-) indicates that data are supressed due to insufficient data (~<10 cases within the strata). Asterisk (*) indicates caution when interpreting data due to a lack of model convergence. Age standardised and fully standardised rates not produced where count is 5 or fewer.

**Supplementary Material 6:** Variation in lung cancer treatment by deprivation, including crude, age-standardised and marginally-standardised results.

|  |  |  |  |  | Odds Ratios (95% CI) | |
| --- | --- | --- | --- | --- | --- | --- |
|  | n | % | Age Adj. % | Fully Adj. % | Crude OR | Adj. OR |
| **NZDep 1-2** |  |  |  |  |  |  |
| Surgery Only | 336 | 15% | 16% | 10% | Ref | Ref |
| Surgery + Radiation | 51 | 2% | 2% | 2% | Ref | Ref |
| Surgery + Systemic Therapy | 75 | 3% | 4% | 3% | Ref | Ref |
| Surgery, Radiation and Systemic Therapy | 34 | 1% | 2% | 2% | Ref | Ref |
|  |  |  |  |  |  |  |
| Radiation Only | 507 | 22% | 21% | 21% | Ref | Ref |
| Radiation + Systemic Therapy | 349 | 15% | 19% | 21% | Ref | Ref |
|  |  |  |  |  |  |  |
| Systemic Therapy Only | 197 | 9% | 10% | 10% | Ref | Ref |
|  |  |  |  |  |  |  |
| **NZDep 3-4** |  |  |  |  |  |  |
| Surgery Only | 321 | 12% | 13% | 8% | 0.81 (0.69-0.96) | 0.75 (0.60-0.94) |
| Surgery + Radiation | 44 | 2% | 2% | 2% | 0.75 (0.50-1.13) | 0.78 (0.51-1.18) |
| Surgery + Systemic Therapy | 88 | 3% | 4% | 3% | 1.03 (0.75-1.41) | 1.19 (0.86-1.64) |
| Surgery, Radiation and Systemic Therapy | 40 | 2% | 2% | 2% | 1.03 (0.65-1.64) | 1.09 (0.68-1.74) |
|  |  |  |  |  |  |  |
| Radiation Only | 538 | 21% | 20% | 20% | 0.91 (0.80-1.05) | 0.92 (0.79-1.06) |
| Radiation + Systemic Therapy | 390 | 15% | 20% | 21% | 0.98 (0.83-1.14) | 1.00 (0.84-1.19) |
|  |  |  |  |  |  |  |
| Systemic Therapy Only | 220 | 8% | 10% | 10% | 0.98 (0.80-1.19) | 0.99 (0.80-1.22) |
|  |  |  |  |  |  |  |
| **NZDep 5-6** |  |  |  |  |  |  |
| Surgery Only | 469 | 14% | 15% | 9% | 0.90 (0.78-1.05) | 0.91 (0.74-1.13) |
| Surgery + Radiation | 56 | 2% | 2% | 2% | 0.72 (0.49-1.05) | 0.74 (0.50-1.10) |
| Surgery + Systemic Therapy | 102 | 3% | 4% | 3% | 0.89 (0.66-1.21) | 1.06 (0.77-1.46) |
| Surgery, Radiation and Systemic Therapy | 46 | 1% | 2% | 2% | 0.89 (0.57-1.39) | 0.94 (0.59-1.48) |
|  |  |  |  |  |  |  |
| Radiation Only | 774 | 22% | 21% | 21% | 1.00 (0.88-1.14) | 1.01 (0.88-1.15) |
| Radiation + Systemic Therapy | 490 | 14% | 19% | 20% | 0.91 (0.78-1.06) | 0.92 (0.78-1.09) |
|  |  |  |  |  |  |  |
| Systemic Therapy Only | 293 | 8% | 10% | 10% | 0.98 (0.81-1.18) | 0.99 (0.81-1.21) |
|  |  |  |  |  |  |  |
| **NZDep 7-8** |  |  |  |  |  |  |
| Surgery Only | 507 | 11% | 12% | 9% | 0.75 (0.65-0.87) | 0.87 (0.71-1.07) |
| Surgery + Radiation | 75 | 2% | 2% | 2% | 0.75 (0.53-1.08) | 0.83 (0.57-1.21) |
| Surgery + Systemic Therapy | 81 | 2% | 2% | 2% | 0.55 (0.40-0.75) | 0.72 (0.51-1.00) |
| Surgery, Radiation and Systemic Therapy | 56 | 1% | 2% | 2% | 0.85 (0.55-1.30) | 0.90 (0.57-1.40) |
|  |  |  |  |  |  |  |
| Radiation Only | 1042 | 24% | 23% | 23% | 1.08 (0.96-1.22) | 1.12 (0.98-1.27) |
| Radiation + Systemic Therapy | 602 | 14% | 18% | 18% | 0.87 (0.76-1.01) | 0.83 (0.71-0.98) |
|  |  |  |  |  |  |  |
| Systemic Therapy Only | 344 | 8% | 9% | 9% | 0.89 (0.74-1.07) | 0.89 (0.73-1.08) |
|  |  |  |  |  |  |  |
| **NZDep 9-10** |  |  |  |  |  |  |
| Surgery Only | 518 | 10% | 10% | 8% | 0.63 (0.54-0.73) | 0.77 (0.62-0.95) |
| Surgery + Radiation | 59 | 1% | 1% | 1% | 0.49 (0.34-0.72) | 0.55 (0.36-0.83) |
| Surgery + Systemic Therapy | 140 | 3% | 3% | 3% | 0.80 (0.60-1.06) | 1.02 (0.75-1.41) |
| Surgery, Radiation and Systemic Therapy | 80 | 2% | 2% | 2% | 1.02 (0.68-1.52) | 0.96 (0.62-1.48) |
|  |  |  |  |  |  |  |
| Radiation Only | 1178 | 22% | 22% | 22% | 1.00 (0.89-1.13) | 1.07 (0.94-1.22) |
| Radiation + Systemic Therapy | 797 | 15% | 18% | 18% | 0.98 (0.86-1.13) | 0.80 (0.68-0.94) |
|  |  |  |  |  |  |  |
| Systemic Therapy Only | 498 | 9% | 10% | 10% | 1.10 (0.93-1.31) | 1.00 (0.82-1.21) |
|  |  |  |  |  |  |  |

**Note:** Age adj. %: age-standardised; Fully adj. % = marginally-standardised. Adj. OR: adjusted for age, sex, ethnicity, rurality, comorbidity, tumour type and stage. Dash (-) indicates that data are supressed due to insufficient data (~<10 cases within the strata). Asterisk (*) indicates caution when interpreting data due to a lack of model convergence. Age standardised and fully standardised rates not produced where count is 5 or fewer.

**Supplementary Material 7:** Variation in lung cancer treatment by rurality, including crude, age-standardised and marginally-standardised results.

|  |  |  |  |  | Odds Ratios (95% CI) | |
| --- | --- | --- | --- | --- | --- | --- |
|  | n | % | Age Adj. % | Fully Adj. % | Crude OR | Adj. OR |
| **Urban 1** |  |  |  |  |  |  |
| Surgery Only | 1311 | 14% | 14% | 9% | Ref | Ref |
| Surgery + Radiation | 161 | 2% | 2% | 1% | Ref | Ref |
| Surgery + Systemic Therapy | 303 | 3% | 4% | 3% | Ref | Ref |
| Surgery, Radiation and Systemic Therapy | 134 | 1% | 2% | 1% | Ref | Ref |
|  |  |  |  |  |  |  |
| Radiation Only | 2117 | 22% | 21% | 22% | Ref | Ref |
| Radiation + Systemic Therapy | 1386 | 14% | 18% | 18% | Ref | Ref |
|  |  |  |  |  |  |  |
| Systemic Therapy Only | 797 | 8% | 9% | 10% | Ref | Ref |
|  |  |  |  |  |  |  |
| **Urban 2** |  |  |  |  |  |  |
| Surgery Only | 374 | 9% | 10% | 8% | 0.66 (0.59-0.75) | 0.77 (0.65-0.91) |
| Surgery + Radiation | 56 | 1% | 1% | 1% | 0.85 (0.62-1.15) | 0.99 (0.72-1.36) |
| Surgery + Systemic Therapy | 86 | 2% | 3% | 3% | 0.69 (0.54-0.87) | 0.93 (0.72-1.20) |
| Surgery, Radiation and Systemic Therapy | 50 | 1% | 2% | 2% | 0.91 (0.66-1.26) | 1.11 (0.79-1.56) |
|  |  |  |  |  |  |  |
| Radiation Only | 892 | 22% | 22% | 22% | 1.04 (0.95-1.13) | 0.96 (0.87-1.05) |
| Radiation + Systemic Therapy | 569 | 14% | 19% | 18% | 1.00 (0.90-1.11) | 1.01 (0.89-1.14) |
|  |  |  |  |  |  |  |
| Systemic Therapy Only | 360 | 9% | 10% | 11% | 1.11 (0.98-1.27) | 1.17 (1.02-1.35) |
|  |  |  |  |  |  |  |
| **Rural 1** |  |  |  |  |  |  |
| Surgery Only | 300 | 11% | 12% | 9% | 0.78 (0.68-0.89) | 0.98 (0.82-1.18) |
| Surgery + Radiation | 45 | 2% | 2% | 2% | 0.98 (0.70-1.36) | 1.08 (0.76-1.52) |
| Surgery + Systemic Therapy | 58 | 2% | 3% | 2% | 0.66 (0.50-0.88) | 0.77 (0.57-1.04) |
| Surgery, Radiation and Systemic Therapy | 38 | 1% | 2% | 2% | 0.99 (0.69-1.42) | 1.10 (0.75-1.60) |
|  |  |  |  |  |  |  |
| Radiation Only | 668 | 24% | 23% | 23% | 1.14 (1.03-1.25) | 1.05 (0.94-1.17) |
| Radiation + Systemic Therapy | 419 | 15% | 19% | 18% | 1.07 (0.95-1.20) | 1.00 (0.87-1.14) |
|  |  |  |  |  |  |  |
| Systemic Therapy Only | 249 | 9% | 10% | 10% | 1.10 (0.95-1.28) | 1.10 (0.94-1.29) |
|  |  |  |  |  |  |  |
| **Rural 2** |  |  |  |  |  |  |
| Surgery Only | 132 | 10% | 10% | 8% | 0.68 (0.57-0.83) | 0.75 (0.58-0.97) |
| Surgery + Radiation | 20 | 1% | 1% | 1% | 0.88 (0.55-1.40) | 0.90 (0.55-1.45) |
| Surgery + Systemic Therapy | 29 | 2% | 3% | 2% | 0.67 (0.46-0.99) | 0.83 (0.55-1.25) |
| Surgery, Radiation and Systemic Therapy | 25 | 2% | 2% | 2% | 1.33 (0.86-2.05) | 1.51 (0.96-2.37) |
|  |  |  |  |  |  |  |
| Radiation Only | 308 | 23% | 22% | 21% | 1.04 (0.91-1.19) | 0.94 (0.81-1.08) |
| Radiation + Systemic Therapy | 209 | 15% | 19% | 19% | 1.08 (0.92-1.27) | 1.06 (0.89-1.27) |
|  |  |  |  |  |  |  |
| Systemic Therapy Only | 122 | 9% | 9% | 11% | 1.09 (0.90-1.34) | 1.12 (0.90-1.38) |
|  |  |  |  |  |  |  |
| **Rural 3** |  |  |  |  |  |  |
| Surgery Only | 34 | 13% | 12% | 9% | 0.96 (0.67-1.38) | 1.03 (0.63-1.69) |
| Surgery + Radiation | 3 | 1% | - | - | 0.69 (0.22-2.18) | 0.75 (0.23-2.39) |
| Surgery + Systemic Therapy | 10 | 4% | 4% | 3% | 1.24 (0.65-2.35) | 1.14 (0.58-2.25) |
| Surgery, Radiation and Systemic Therapy | 9 | 3% | 4% | 3% | 2.55 (1.29-5.07) | 2.49 (1.22-5.11) |
|  |  |  |  |  |  |  |
| Radiation Only | 54 | 21% | 21% | 20% | 0.94 (0.69-1.27) | 0.88 (0.64-1.22) |
| Radiation + Systemic Therapy | 45 | 17% | 20% | 21% | 1.25 (0.90-1.74) | 1.22 (0.85-1.75) |
|  |  |  |  |  |  |  |
| Systemic Therapy Only | 24 | 9% | 10% | 11% | 1.13 (0.74-1.74) | 1.12 (0.72-1.75) |
|  |  |  |  |  |  |  |

**Note:** Age adj. %: age-standardised; Fully adj. % = marginally-standardised. Adj. OR: adjusted for age, sex, ethnicity, deprivation, comorbidity, tumour type and stage. Dash (-) indicates that data are supressed due to insufficient data (~<10 cases within the strata). Asterisk (*) indicates caution when interpreting data due to a lack of model convergence. Age standardised and fully standardised rates not produced where count is 5 or fewer.

**Supplementary Material 8:** Variation in lung cancer treatment by comorbidity, including crude, age-standardised and marginally-standardised results.

|  |  |  |  |  | Odds Ratios (95% CI) | |
| --- | --- | --- | --- | --- | --- | --- |
| **C3 Comorbidity Index Score** | n | % | Age Adj. % | Fully Adj. % | Crude OR | Adj. OR |
| **<=0** |  |  |  |  |  |  |
| Surgery Only | 1031 | 12% | 12% | 8% | Ref | Ref |
| Surgery + Radiation | 140 | 2% | 2% | 1% | Ref | Ref |
| Surgery + Systemic Therapy | 305 | 4% | 4% | 3% | Ref | Ref |
| Surgery, Radiation and Systemic Therapy | 155 | 2% | 2% | 2% | Ref | Ref |
|  |  |  |  |  |  |  |
| Radiation Only | 1939 | 22% | 22% | 22% | Ref | Ref |
| Radiation + Systemic Therapy | 1609 | 19% | 21% | 21% | Ref | Ref |
|  |  |  |  |  |  |  |
| Systemic Therapy Only | 906 | 10% | 11% | 11% | Ref | Ref |
|  |  |  |  |  |  |  |
| **0-1** |  |  |  |  |  |  |
| Surgery Only | 374 | 15% | 16% | 9% | 1.30 (1.15-1.48) | 1.09 (0.91-1.30) |
| Surgery + Radiation | 54 | 2% | 2% | 2% | 1.34 (0.97-1.84) | 1.22 (0.89-1.69) |
| Surgery + Systemic Therapy | 91 | 4% | 5% | 3% | 1.03 (0.81-1.31) | 1.10 (0.86-1.42) |
| Surgery, Radiation and Systemic Therapy | 51 | 2% | 3% | 2% | 1.14 (0.83-1.57) | 1.28 (0.92-1.77) |
|  |  |  |  |  |  |  |
| Radiation Only | 579 | 23% | 20% | 23% | 1.04 (0.93-1.16) | 1.04 (0.93-1.16) |
| Radiation + Systemic Therapy | 376 | 15% | 20% | 20% | 0.77 (0.68-0.87) | 0.94 (0.82-1.07) |
|  |  |  |  |  |  |  |
| Systemic Therapy Only | 211 | 8% | 9% | 10% | 0.79 (0.67-0.92) | 0.89 (0.76-1.05) |
|  |  |  |  |  |  |  |
| **1-2** |  |  |  |  |  |  |
| Surgery Only | 313 | 12% | 13% | 9% | 0.97 (0.85-1.12) | 1.08 (0.89-1.29) |
| Surgery + Radiation | 45 | 2% | 2% | 2% | 1.03 (0.74-1.45) | 1.14 (0.81-1.61) |
| Surgery + Systemic Therapy | 46 | 2% | 2% | 2% | 0.48 (0.35-0.65) | 0.61 (0.44-0.84) |
| Surgery, Radiation and Systemic Therapy | 26 | 1% | 1% | 1% | 0.53 (0.35-0.81) | 0.69 (0.45-1.06) |
|  |  |  |  |  |  |  |
| Radiation Only | 596 | 22% | 22% | 22% | 0.98 (0.89-1.09) | 0.98 (0.88-1.09) |
| Radiation + Systemic Therapy | 353 | 13% | 17% | 18% | 0.66 (0.58-0.75) | 0.83 (0.72-0.95) |
|  |  |  |  |  |  |  |
| Systemic Therapy Only | 206 | 8% | 9% | 10% | 0.71 (0.60-0.83) | 0.84 (0.71-0.98) |
|  |  |  |  |  |  |  |
| **>2** |  |  |  |  |  |  |
| Surgery Only | 436 | 10% | 13% | 9% | 0.85 (0.76-0.96) | 1.25 (1.06-1.48) |
| Surgery + Radiation | 46 | 1% | 1% | 1% | 0.67 (0.48-0.94) | 0.85 (0.60-1.21) |
| Surgery + Systemic Therapy | 44 | 1% | 2% | 2% | 0.29 (0.21-0.40) | 0.49 (0.35-0.68) |
| Surgery, Radiation and Systemic Therapy | 24 | 1% | 1% | 1% | 0.31 (0.20-0.48) | 0.53 (0.34-0.83) |
|  |  |  |  |  |  |  |
| Radiation Only | 928 | 22% | 24% | 22% | 0.98 (0.89-1.07) | 0.97 (0.88-1.07) |
| Radiation + Systemic Therapy | 291 | 7% | 11% | 13% | 0.32 (0.28-0.37) | 0.49 (0.43-0.57) |
|  |  |  |  |  |  |  |
| Systemic Therapy Only | 230 | 5% | 7% | 8% | 0.49 (0.42-0.57) | 0.68 (0.58-0.79) |
|  |  |  |  |  |  |  |

**Note:** Age adj. %: age-standardised; Fully adj. % = marginally-standardised. Adj. OR: adjusted for age, sex, ethnicity, deprivation, rurality, tumour type and stage. Dash (-) indicates that data are supressed due to insufficient data (~<10 cases within the strata). Asterisk (*) indicates caution when interpreting data due to a lack of model convergence. Age standardised and fully standardised rates not produced where count is 5 or fewer.
